# « Transsexualism » or « gender dysphoria » : what impact on psychological well-being ?

**DOI:** 10.1192/j.eurpsy.2025.2315

**Published:** 2025-08-26

**Authors:** N. Regaieg, F. Guermazi, F. Cherif, D. Mnif, A. Jmal, M. Ben Jemaa, I. Baati, J. Masmoudi

**Affiliations:** 1 Psychiatry A department, Hedi Chaker University Hospital; 2 Family medicine department, University of Sfax, Faculty of Medicine of Sfax; 3 Community Health and Epidemiology Department, Hedi Chaker University Hospital, Sfax, Tunisia

## Abstract

**Introduction:**

Gender dysphoria is the feeling of discomfort or distress that might occur in people whose gender identity differs from their sex assigned at birth or sex-related physical characteristics. Individuals with gender dysphoria frequently face social as well as psychoaffective difficulties that can impede their well-being and quality of life.

**Objectives:**

The aim of this study were to assess the impact of gender dysphoria among young medical trainees on their psychological health in terms of stress, anxiety and depression.

**Methods:**

A cross-sectional, descriptive, and analytical study was conducted with a Tunisian population of young medical trainees, during the period of time from October 1, 2023, to January 31, 2024. Data were collected using a questionnaire created with GOOGLE FORMS including an information sheet and two psychometric assessment tools : the Depression, Anxiety, and Stress Scale (DASS-21) and the gender identity/gender dysphoria questionnaire for adolescents and adults (GIDYQ-AA) asssessing subjective, somatic, social, and sociolegal aspects.

**Results:**

A total of 111 participants took part in this study. Their median age was 28 years. They were single in 56.6% of cases, with a male-to-female ratio of 0.56.

The prevalence of depression, anxiety, and stress was 53.2%, 59.5%, and 34.2%, respectively.

Median scores of depresssion, anxiety and stress were 10 (IQR =[2–18]), 8 (IQR =[4–14]) and 10 (IQR =[6–20]), respectively.

The overall median score on the GIDYQ-AA scale was 4.85 (IQR =[4.77–5.0]). The social dimension had the lowest median score at 4.88 (IQR=[4.55–5.0]) while the median score of the subjective dimension was 4.92 (IQR=[4.69-5.0]). Somatic and socio-legal median scores were 5.0 (IQR=[5.0–5.0]).

The score for the subjective dimension of the GIDYQ-AA was negatively correlated with anxiety (p=0.04) and stress (p=0.04) scores.

**Conclusions:**

The psychological vulnerability of young medical trainees may be exacerbated by intrapsychic conflicts which may be related to their gender identity. It is essential to identify and consider the psychological factors associated with gender dysphoria in the care pathway of these individuals, through appropriate psychiatric evaluation and support in order to better guide therapeutic decisions regarding sex reassignment.

**Disclosure of Interest:**

None Declared

